# Chemical and Genetic Modulation of Complex I of the Electron Transport Chain Enhances the Biotherapeutic Protein Production Capacity of CHO Cells

**DOI:** 10.3390/cells12222661

**Published:** 2023-11-20

**Authors:** Corey Kretzmer, Kelsey Reger, Vincent Balassi, Quang Long Pham, Michael Johns, Samuel T. Peters, Amber Petersen, Jana Mahadevan, Jason Gustin, Trissa Borgschulte, David Razafsky

**Affiliations:** Upstream Research and Development, MilliporeSigma, Saint Louis, MO 63103, USAamber.petersen@milliporesigma.com (A.P.); jason.gustin@milliporesigma.com (J.G.); trissa.borgschulte@milliporesigma.com (T.B.)

**Keywords:** Chinese hamster ovary (CHO), cell line engineering, bioprocessing, perfusion, recombinant protein, small molecules, mitochondria, Ndufa13, electron transport chain

## Abstract

Chinese hamster ovary (CHO) cells are the cell line of choice for producing recombinant therapeutic proteins. Despite improvements in production processes, reducing manufacturing costs remains a key driver in the search for more productive clones. To identify media additives capable of increasing protein production, CHOZN^®^ GS^−/−^ cell lines were screened with 1280 small molecules, and two were identified, forskolin and BrdU, which increased productivity by ≥40%. While it is possible to incorporate these small molecules into a commercial-scale process, doing so may not be financially feasible or could raise regulatory concerns related to the purity of the final drug substance. To circumvent these issues, RNA-Seq was performed to identify transcripts which were up- or downregulated upon BrdU treatment. Subsequent Reactome pathway analysis identified the electron transport chain as an affected pathway. CRISPR/Cas9 was utilized to create missense mutations in two independent components of the electron transport chain and the resultant clones partially recapitulated the phenotypes observed upon BrdU treatment, including the productivity of recombinant therapeutic proteins. Together, this work suggests that BrdU can enhance the productivity of CHO cells by modulating cellular energetics and provides a blueprint for translating data from small molecule chemical screens into genetic engineering targets to improve the performance of CHO cells. This could ultimately lead to more productive host cell lines and a more cost-effective method of supplying medication to patients.

## 1. Introduction

The Chinese hamster ovary (CHO) cell is an industry-leading cell line for the production of therapeutic proteins [1,2]. This is largely due to its advantageous manufacturing characteristics and the wealth of experience that the industry has accumulated over the past several decades. The manufacturing process starts with the development of a single cell clone with favorable manufacturing phenotypes, most notably the ability to secrete large quantities of a therapeutic protein. Historically, obtaining a high-expressing, stable clone would require numerous rounds of screening, which could take upwards of 9–12 months [1]. As molecular biology techniques have improved, several approaches, including plasmid optimization, the use of ubiquitous chromatin opening elements (UCOEs), incorporation of transposase technologies, and the use of site-specific integration have been used to reduce development timelines, increase productivity, and enhance the probability of obtaining a high-expressing, stable clone [3,4,5,6]. Despite these advances, further improvements must be made to increase production levels and reduce the cost of developing and screening suitable cell lines for drug manufacturing [7].

Several studies indicate that the supplementation of small molecules into cell culture media can increase recombinant protein production, including a 3× increase in monoclonal antibody production, and a nearly 100× increase in the production of fluorescent and luminescent proteins [3,8,9]. Despite these benefits, small molecules are rarely incorporated into industrial-scale processes for several reasons. Doing so may require additional time and equipment to remove the small molecule during manufacturing processes and require the development of an assay to demonstrate sufficient clearance from the final drug substance. Additionally, significant testing would be required to ensure that expensive purification resins remain unchanged and available for cleaning and reuse in future production batches. Finally, the stability, availability, and quality of the new raw material would need to be carefully assessed, leading to substantial time and costs in the production of a therapeutic protein. Although these factors present significant hurdles for the incorporation of small molecules into manufacturing processes, valuable information can still be gained about the underlying biology of CHO cells through treatment with small molecules. Importantly, this information could lead towards the genetic engineering of a more productive CHO cell and reduce the amount of screening required to identify a high-producing clone during the cell line development process.

In this study, we used a pre-assembled small molecule library (LOPAC^®1280^) containing 1280 pharmacologically active compounds and identified two compounds, the plant-derived labdane diterpene forskolin and the synthetic thymidine analog BrdU (5-bromo-2′-deoxyuridine), which enhanced the productivity of four independently derived clonal cell lines by ≥40%. Interestingly, the benefits of supplementing BrdU into the media were observed in not only stably expressing cell lines, but also in a transient protein production system. While forskolin has been shown to induce cell cycle arrest via the LKB1/AMPK pathway, inhibit apoptosis via the CREB/Bcl-2 pathway, and ultimately increase cell-specific productivity of CHO cells expressing a monoclonal antibody, the effects of BrdU are not well-understood [10]. Since adding a pharmacologically active compound to a production process may not be a favorable path to enhance protein production, we performed differential gene expression analysis to determine which cellular pathways were being modulated by the addition of BrdU. We identified 24 genes with decreased expression and 341 genes with increased expression upon treatment with BrdU (genes with at least a log_2_FC of 2 and a *p*-value < 0.05 were considered significant). Further analysis of the Reactome pathways revealed that components of the electron transport chain and ribosome were downregulated. In an effort to determine if modulation of the electron transport chain had a similar effect as BrdU treatment, we utilized the CRISPR/Cas9 system to reduce the expression of *Ndufa13*, a subunit of the electron transport chain Respiratory Complex I, and were able to recapitulate ~25% of the cell-specific productivity benefits associated with adding BrdU to the media. In fact, when comparing the performance of *Ndufa13* edited and unedited clones, the top seven clones, as ranked by cell-specific productivity, came from the edited population. We anticipate that incorporating further genetic changes of targets identified in the differential gene expression analysis into the *Ndufa13* edited clones would likely lead to further phenotypic enhancements, as was observed in BrdU-treated cultures. This gene editing strategy could play an important role in enhancing the efficiency of manufacturing therapeutic proteins without the drawbacks associated with adding small molecules to the media.

## 2. Materials and Methods

### 2.1. LOPAC^®1280^ Chemical Library Screen

The LOPAC^®1280^ chemical library (Sigma Aldrich, St. Louis, MO, USA) was provided pre-solubilized in DMSO at 10 mM. Four model CHOZN^®^ GS^−/−^ cell lines were seeded in EX-CELL^®^ CD Fusion media (Sigma Aldrich, St. Louis, MO, USA) at 2.5 × 10^4^ viable cells/mL into 96-well non-tissue culture-treated plates (Greiner, Kremsmunster, Austria). Small molecules from the LOPAC^®1280^ library were diluted in DMSO (Sigma Aldrich), then administered to cell culture plates at final concentrations of 3 µM and 30 µM, such that each well received a single compound. Untreated and vehicle-treated cultures were maintained as controls by administering library-equivalent volumes of media or DMSO, respectively. Plates were maintained in static incubators at 37 °C and 5% CO_2_ for seven days, and outgrowth was monitored by a Cell Metric^®^ (Solentim, Wimborne Minster, UK) plate imager. After seven days, clarified supernatant was collected and analyzed for biotherapeutic protein concentration by ForteBio QKe (Sartorius, Gottingen, Germany).

### 2.2. Transient Transfection

CHOZN^®^ GS^−/−^ cells (Sigma Aldrich, St. Louis, MO, USA) were gradually adapted to CHOgro^®^ Expression Medium (Mirus^®^, Marietta, GA, USA) supplemented with 6 mM L-glutamine solution (Mirus^®^) and 0.3% Poloxamer 188 (Mirus^®^) until consistent viability and growth rates were obtained. CHOgro^®^ Medium adapted-CHOZN^®^ GS^−/−^ cells were cultured in a shaking incubator at 37 °C, 5% CO_2_, and 250 rpm. One day prior to transfection, CHOZN^®^ GS^−/−^ cells were seeded at 1.2 × 10^6^ viable cells/mL in CHOgro^®^ Medium. On the day of transfection, CHOZN^®^ GS^−/−^ cells were counted on a Cellaca^®^ MX High-Throughput Automated Cell Counter (Nexcelom Bioscience, Lawrence, MA, USA) and seeded at 2 × 10^6^ viable cells/mL in CHOgro^®^ Medium at a final culture volume of 3 mL in 24-deep well plates (Axygen, Alachua, FL, USA) or 20 mL in a TubeSpin^®^ Bioreactor. CHOZN^®^ GS^−/−^ cells were transfected in CHOgro^®^ medium following the manufacturer’s recommended protocol. One day post-transfection, 2 × 10^6^ viable cells/mL were seeded in EX-CELL^®^ Advanced CHO Fed-Batch medium (Sigma Aldrich, St. Louis, MO, USA) for a fed-batch assay, in triplicate, in either 24-deep well plates or TubeSpin^®^ Bioreactors. Each well was fed 15% of the initial volume of EX-CELL^®^ Advanced CHO Feed 1 (Sigma Aldrich) and given a supplement of 6 g/L glucose (Sigma Aldrich, St. Louis, MO, USA) and 6 mM L-Glutamine (Sigma Aldrich). BrdU (Sigma Aldrich, St. Louis, MO, USA) was reconstituted at 200 µM directly into the media and all control wells were treated with an equal volume of EX-CELL^®^ Advanced CHO Fed-Batch medium. Samples were then placed into a shaker at 32 °C, 5% CO_2_, and 250 RPM at a 50 mm orbital throw (TubeSpin^®^ Bioreactors) or 320 RPM at a 25 mm orbital throw (24-deep well plates). On day five, cultures were given a bolus of 6 mM L-glutamine and 6 g/L glucose. Cell growth and viability were measured on the Cellaca^®^ every other day starting on day three. Clarified culture supernatant was harvested every other day starting on day three and utilized for titer analysis on the ForteBio Octet QKe (Sartorius, Gottingen, Germany). Metabolite analysis was performed on the NOVA Bioprofile Flex2 (Novabiomedical). Cell cultures were terminated when the viability dropped below 70% or on day 13/day 14.

### 2.3. Batch Assay

BrdU (Sigma Aldrich, St. Louis, MO, USA) and forskolin (Sigma Aldrich, St. Louis, MO, USA) were solubilized in DMSO to 10 mM. Four model CHOZN^®^ GS^−/−^ cell lines were tested in biological triplicates by inoculation with BrdU, forskolin, or volume equivalents of DMSO or media for vehicle and untreated controls, respectively. A standard 7-day batch assay was performed following established protocols in TubeSpin^®^ Bioreactor Tubes (TPP Techno Plastic Products, Trasadingen, Switzerland) in 30 mL of EX-CELL^®^ Advanced CHO Fed-batch Medium (Sigma Aldrich, St. Louis, MO, USA). Briefly, cultures were seeded at 3 × 10^5^ viable cells/mL and cultured at 200 RPM and 50 mM throw for seven days at 37 °C, 5% CO_2_, and 80% humidity. On days three and five, cultures were fed 5 g/L glucose (Sigma Aldrich, St. Louis, MO, USA), and cultures were counted on days three, four, six, and seven on a Vi-CELL^™^ XR Cell Viability Analyzer (Beckmann Coulter, Brea, CA, USA) to monitor growth. After seven days, clarified supernatant was collected and analyzed for biotherapeutic protein concentration by ForteBio Octet QKe (Sartorius, Gottingen, Germany). Cell-specific productivity was calculated by taking the volumetric titer and dividing by the integral viable cell density (IVCD), also known as the total working viable cell density, over the course of the assay.

### 2.4. Simulated Perfusion Assay

BrdU was solubilized in DMSO at 250 mM. Model CHOZN^®^ GS^−/−^ cell line IgG-B was seeded in biological duplicates at 1 × 10^7^ viable cells/mL in 20 mL of EX-CELL^®^ Advanced HD Perfusion Medium (Sigma Aldrich, St. Louis, MO, USA) in TubeSpin^®^ Bioreactor Tubes (TPP Techno Plastic Products, Trasadingen, Switzerland). Daily full volume media exchanges were performed by pelleting the cells via centrifugation, aspiration of spent media, then resuspension in fresh media containing either 1 mM BrdU or volume-equivalent DMSO as a control. Clarified media were saved for titer analysis by ForteBio Octet QKe (Sartorius, Gottingen, Germanny) and for metabolite analysis on a NOVA Bioprofile Flex2 (Novabiomedical, Waltham, MA, USA). Cultures were monitored through daily counts by Vi-CELL^™^ XR Cell Viability Analyzer (Beckmann Coulter, Brea, CA, USA). Daily cell-specific productivity was calculated by dividing Day(N) volumetric titer by the averaged VCD of Day(N) and Day(N − 1).

### 2.5. RNA Sequencing and Analysis

RNA was isolated in biological duplicate from 5 × 10^6^ viable cells on day 4 of a simulated perfusion assay using the RNeasy mini kit (Qiagen, Hilden, Germany). Total RNA integrity was determined using an Agilent Bioanalyzer. Library preparation was performed with 5 to 10 ug of total RNA with a Bioanalyzer RIN score greater than 8.0. Ribosomal RNA was removed by poly-A selection using Oligo-dT beads (mRNA Direct kit, Life Technologies). mRNA was then fragmented in reverse transcriptase buffer and heated to 94 °C for 8 min. mRNA was reverse transcribed to yield cDNA using SuperScript III RT enzyme per the manufacturer’s instructions (Life Technologies, Carlsbad, CA, USA) and random hexamers. A second strand reaction was performed to yield ds-cDNA. cDNA was blunt-ended, had an A base added to the 3′ ends, and Illumina sequencing adapters were ligated to the ends. Ligated fragments were then amplified for 12–15 cycles using primers incorporating unique dual index tags. Fragments were sequenced on an Illumina NovaSeq-6000 using PE150 reads.

Basecalls and demultiplexing were performed with Illumina’s bcl2fastq software and a custom python demultiplexing program with a maximum of one mismatch in the indexing read. RNA-seq reads were then aligned to the Ensembl release 99 *Cricetulus griseus* (CriGri_1.0.99) primary assembly with STAR version 2.5.1a [11]. Gene counts were derived from the number of uniquely aligned unambiguous reads by Subread:featureCount version 1.4.6-p5 [12]. Isoform expression of known Ensembl transcripts was estimated with Salmon version 0.8.2 [13]. Sequencing performance was assessed for the total number of aligned reads, total number of uniquely aligned reads, and features detected. The ribosomal fraction, known junction saturation, and read distribution over known gene models were quantified with RSeQC version 2.6.2 [14].

All gene counts were then imported into the R/Bioconductor package EdgeR and TMM normalization size factors were calculated to adjust for samples with differences in library size [15]. Ribosomal genes and genes not expressed in the smallest group size minus one sample greater than one count per million were excluded from further analysis. The TMM size factors and the matrix of counts were then imported into the R/Bioconductor package Limma [16]. Weighted likelihoods based on the observed mean–variance relationship of every gene and sample were then calculated for all samples and the count matrix was transformed to moderated log_2_ counts per million with Limma’s voomWithQualityWeights [17]. The performance of all genes was assessed with plots of the residual standard deviation of every gene to their average log-count with a robustly fitted trend line of the residuals. Differential expression analysis was then performed using a published CHO genome assembly [18] consisting of 20,414 annotated genes, of which 13,323 had ≥2 mapped reads in this analysis, to analyze for differences between conditions, and the results were filtered for only those genes with Benjamini–Hochberg false discovery rate-adjusted *p*-values less than or equal to 0.05. Reactome pathway analysis was performed via the CHOmics web-based platform [19].

To determine the rate of mutations in BrdU and control samples, adapters were trimmed from the fastq sequences with cutadapt using a minimum read length of 20. Reads were mapped to the Cri-Gri_PICRH1.0 genome [20], the mitochondrial sequence from the CriGri_1.0 genome [21], and the IgG heavy and light chain sequences using the STAR 2-pass workflow with mapping quality adjusted to 60. Read pileups were generated for the uniquely mapped reads aligning to the IgG heavy and light chains (mapping quality: MQ = 60) in each bam file using bcftools mpileup with no read downsampling (max read depth set to 100,000,000 -max-depth 100,000,000). Variants were then called from the read pileups using bcftools multiallelic caller (bcftools call -m) and a custom ploidy file containing integration copy numbers of IgG heavy and light chains [22]. Variant quality was assessed. The high-quality base calls were extracted from the DP4 field in each vcf file to obtain reference and non-reference base calls for each position of the IgG heavy and light chains. Overall mutation rates were identified using the total number of base calls that matched or mismatched the reference sequence for each condition.

### 2.6. Mitochondrial Mass

Staining solution was prepared by diluting MitoView Green (Biotium, San Francisco, CA, USA) and DAPI (Sigma Aldrich) to final concentrations of 50 nM and 300 nM, respectively, in 1× PBS (Sigma Aldrich). On day four of the simulated perfusion assays, 90 µL of staining solution was mixed with 10 µL of cell culture sample in a 96-well V-bottom plate. The plate was covered with parafilm and placed on a shaker at 120 RPM for 20 min at 37 °C in a humidity-controlled incubator. Cells were analyzed on an iQue3 flow cytometer (Sartorius) by quantifying the mean fluorescence intensity of 523 nm light.

### 2.7. Mitochondrial Membrane Potential (MMP)

*Ndufa13* edited clones, wildtype clones, and cultures treated with BrdU or DMSO were assessed for MMP with Image-IT TMRM reagent (tetramethylrhodamine, methyl ester, perchlorate) (ThermoFisher, Waltham, MA, USA) following the manufacturer’s instructions. Briefly, a staining solution of 100 nM TMRM and 300 nM DAPI (Sigma Aldrich, St. Louis, MO, USA) was diluted in PBS (Sigma Aldrich, St. Louis, MO, USA). Cell culture (10 µL) was mixed with the staining solution (190 µL). Samples were gently shaken in a dark incubator at 37 °C for 30 min. The mean fluorescence intensity of TMRM staining of live cells, as determined via DAPI staining, was quantified at 572 nm on an iQue3 (Sartorius, Gottingen, Germany).

### 2.8. Real-Time ATP Rate Assay

Cellular oxygen consumption rate (OCR), mitochondrial, glycolytic, and total ATP production rate were measured using the Agilent Real-Time ATP Rate Assay Kit (Agilent) on the Seahorse XFe96 Analyzer. The day prior to use, a FluxPak sensor cartridge (Agilent, Santa Clara, CA, USA) was hydrated by placing the sensor probes in 200 µL of Agilent Seahorse XF Calibrant (Agilent, Santa Clara, CA, USA) and placing the sensors in an incubator set to 37 °C and 0% CO_2_. The day of measurement, fresh Seahorse Medium was formulated by mixing 97 mL of Seahorse XF DMEM medium, pH 7.4 (Agilent), with 1 mL of Seahorse XF 200 mM glutamine solution (Agilent), 1 mL of Seahorse XF 100 mM pyruvate solution (Agilent), and 1 mL of Seahorse XF 1.0 M glucose solution (Agilent). The final assay medium was Seahorse DMEM pH 7.4 + 2 mM glutamine, 1 mM pyruvate, and 10 mM glucose. This medium was placed at 37 °C prior to use. On day four of the simulated perfusion assay, cells were diluted 30× in Seahorse DMEM medium (with added glucose, pyruvate, and glutamine). The 30× diluted cell culture samples were then counted on a Vi-CELL™ XR Cell Viability Analyzer (Beckman Coulter, Brea, CA, USA), centrifuged at 1000 RPM for 5 min, and resuspended at 500,000 cells/mL in 1 mL of Seahorse DMEM medium, pH 7.4, + 2 mM glutamine, 1 mM pyruvate, and 10 mM glucose. One hour prior to performing the Real-Time ATP Rate Assay, cell samples were seeded at 25,000 cells/well in an Agilent Seahorse XF96 cell culture plate that had been previously coated with Poly-L-lysine (Sigma Aldrich #P4707), centrifuged at 200× *g* for 2 min to attach the cells in a confluent monolayer, then 130 µL of warm Seahorse DMEM pH 7.4 + 2 mM glutamine, 1 mM pyruvate, and 10 mM glucose were added to each well and the cell culture plate was placed at 37 °C with 0% CO_2_ for no more than 1 h before analyzing the plate. Following the Real-Time ATP Rate user guide supplied by Agilent, compounds oligomycin and rotenone/antimycin A + Hoechst 33,342 stain (ThermoFisher, Waltham, MA, USA) were loaded into the drug ports A and B, respectively, of a previously hydrated FluxPak sensor cartridge to a final working concentration of 1.5 µM oligomycin and 0.5 µM rotenone/antimycin A plus 2 µM of Hoechst stain. The FluxPak sensor cartridge and the cell plate were loaded into the Seahorse Xfe96 Analyzer and cellular energetics (OCR, Mito, Glyco, and Total ATP rate) were measured. Real-Time ATP Rate Assay data were normalized to cell number as follows. (1) After completion of the Real-time ATP Rate Assay, the cell culture plate was immediately transferred to the BioTek Cytation5 for imaging using high-contrast brightfield imaging. Cell number was counted in the center of the well and total cell number was calculated using the Gen5 software. The total cells/well were then entered into the Seahorse Wave software, and data were normalized to 10,000 cells. (2) Alternatively, the cell culture plate was placed in the Cytation5 and the Seahorse Imagine and Cell Counting software was used to count and calculate the total number of cells per well stained with Hoechst stain. Using the normalization function in the Seahorse Wave software, all the Real-Time ATP Rate Assay data were normalized to 10,000 cells.

### 2.9. Ndufa13 and Ndufa5 Cell Line Engineering and Simulated Perfusion Assay

Single guide RNAs (sgRNAs) were designed against exon 3 of *Ndufa13*. Ribonucleoprotein (RNP) complexes were formed by incubating a 3:1 molar ratio of sgRNA to eSpCas9 (Sigma Aldrich) at room temperature for ten minutes, then transfected into CHOZN^®^ GS^−/−^ cell line Fc Fusion-2 by nucleofection (Lonza). Transfected pools were recovered for two days, genomic DNA (gDNA) was isolated on a QiaCube using a DNeasy Blood and Tissue Kit (Qiagen), and cutting efficiency was determined via a Surveyor Nuclease Detection Assay (IDT). Clones were derived from the highest-efficiency RNP-transfected pool via a Sony FX500 FACS system (Sony). Clonality was verified via imaging on a CellMetric (Solentim, Wimborne Minster, UK) and gDNA was isolated from outgrown clones via QuickExtract (Biosearch™ Technologies, Hoddesdon, UK). Genotypes were determined via amplicon sequencing on an Illumina^®^ MiSeq (Illumina^®^, San Diego, CA, USA). NGS-confirmed clones with a nonsense mutation in *Ndufa13* (referred to as edited clones because only a single allele in each clone was mutated) (n = 44) and wildtype counterparts (n = 58) were assayed for growth and productivity in a 96-deep well plate simulated perfusion assay. Briefly, each edited and wildtype clone was seeded at 1 × 10^7^ viable cells/mL in 400 µL of EX-CELL^®^ Advanced HD Perfusion Medium (Sigma Aldrich) in a 96-deep well plate (ThermoFisher, Waltham, MA, USA). Daily half volume exchanges (0.5 VVD) were performed by centrifuging the cells, removing 200 µL of clarified supernatant, and adding 200 µL of fresh EX-CELL^®^ Advanced HD Perfusion Medium (Sigma Aldrich) to the resuspended cells. Culture growth and viability were monitored daily by iQue3 (Sartorius) and protein titer was quantified on a ForteBio Octet QKe (Sartorius).

For *Ndufa5,* gRNAs targeting exon 2 were designed and delivered via lentivirus (Sigma Aldrich) which also harbored the Cas9 coding sequence. Alternatively, a non-targeting control gRNA was utilized to develop wildtype control clones. Pools were selected with 10 ug/mL puromycin (Sigma Aldrich), scaled up, and banked upon recovery. Cells were cloned via a Sony FX500 FACS system (Sony, San Jose, CA, USA) and clonality was verified via imaging on a CellMetric (Solentim, Wilborn Minister, UK). gDNA was isolated from outgrown clones via QuickExtract (Biosearch™ Technologies, Hoddesdon, UK). Genotypes were determined via amplicon sequencing on an Illumina^®^ MiSeq (Illumina^®^, San Diego, CA, USA). Edited and wildtype clones were seeded at 1 × 10^7^ viable cells/mL in 10 mL of EX-CELL^®^ Advanced HD Perfusion Medium (Sigma Aldrich, St. Louis, MO, USA) in TubeSpin^®^ Bioreactor Tubes (TPP Techno Plastic Products, Trasadingen, Switzerland). Daily half volume exchanges (0.5 VVD) were performed by centrifuging the cells, removing 5 mL of clarified supernatant, and adding 5 mL of fresh EX-CELL^®^ Advanced HD Perfusion Medium (Sigma Aldrich, St. Louis, MO, USA) to the resuspended cells. Culture growth and viability were monitored daily on a Vi-CELL™ XR (Beckman, Brea, CA, USA) and protein titer was quantified on a ForteBio Octet QKe (Sartorius, Gottingen, Germany).

### 2.10. Perfusion Culture in 2 mL Mobius^®^ Breez Microbioreactor Systems

Detailed descriptions of the Mobius^®^ Breez system (Sigma Aldrich, St. Louis, MO, USA) with relevant methodologies have been previously described [23]. Prior to performing the automated pH calibration procedure, EX-CELL^®^ Advanced HD Perfusion Medium (Sigma Aldrich, St. Louis, MO, USA) was characterized to determine the relationship between pH and dissolved CO_2_. Reagent bottles were filled with media, water, and 7.5% sodium bicarbonate and cassettes were mounted onto controller pods. After priming inlet lines with reagents, culture chambers were filled with EX-CELL^®^ Advanced HD Perfusion Medium (Sigma Aldrich, St. Louis, MO, USA). The pH, optical density (OD), and dissolved oxygen (DO) sensors were automatically calibrated. Cells were expanded in Cellvento^®^ 4CHO-X Expansion Medium (Sigma Aldrich, St. Louis, MO, USA) using shake flasks in an incubator (Multitron, INFORS HT, Bottmingen, Switzerland) set at 37 °C, 80% humidity, 5% CO_2_, 40% O_2_, and 90 RPM. During expansion, cells were passaged every three or four days. Prior to inoculation, expanded cells were spun down using a centrifuge, supernatant was discarded, and cells were resuspended in 20 mL of EX-CELL^®^ Advanced HD Perfusion Medium. The inoculum was transferred to a 125 mL PETG transfer bottle (Sanisure, Camarillo, CA, USA), the transfer bottle was welded to the inoculation line of the Mobius^®^ Breez system, and inoculation was gently performed via gravity flow. Culture parameters were set at 37 °C, 40% DO, pH 7.00 ± 0.02, and 5 Hz mixing frequency. One hour after inoculation, ~100 µL of sample was taken for cell counts. Perfusion was initiated on day zero and set at ≥1 vessel volume per day (VVD). Daily sampling included 150 µL of bioreactor culture volume which was used for cell counts and clarified by centrifugation for bioreactor titer measurement; in addition, 1.5 mL of harvest volume was collected. Cell counts were performed on a ViCELL-XR (Beckman Coulter, Brea, CA, USA) with a dilution factor of 10× or 15× depending on cell density. Metabolites and osmolality were quantified from the harvest sample using a NOVA Bioprofile Flex2 (Novabiomedical). Protein titers of both bioreactor and harvest samples were quantified on a ForteBio Octet QKe (Sartorius, Gottingen, Germany).

## 3. Results

### 3.1. Identification and Validation of Pharmacologically Active Chemicals That Enhance Therapeutic Protein Production

**Figure 1:**
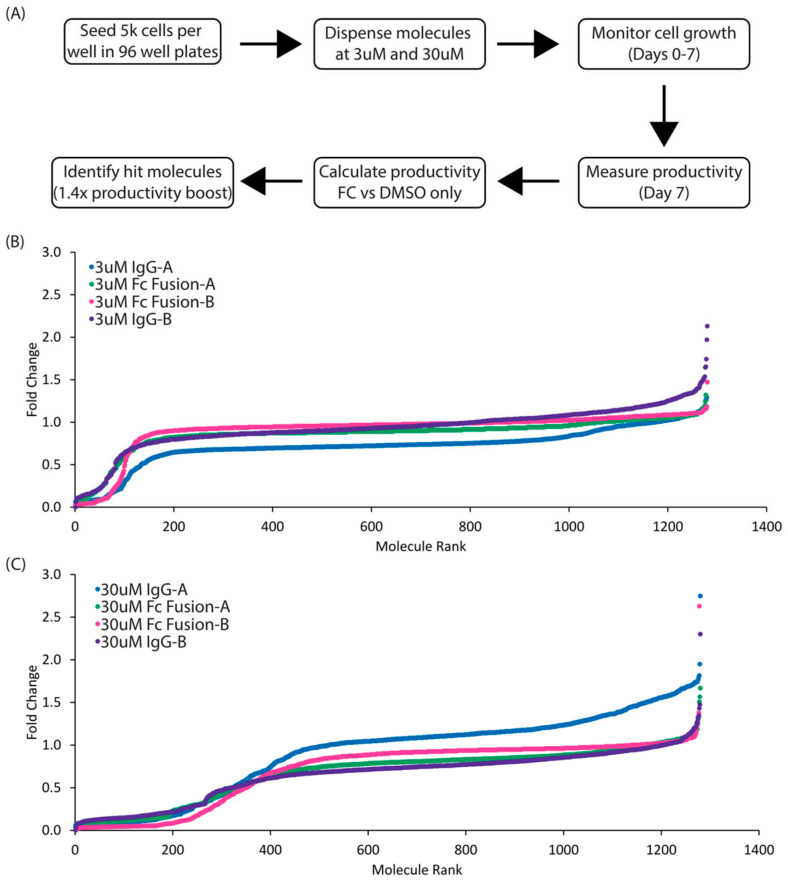
High-throughput screen to enhance recombinant protein production. (**A**) High-throughput screening process to identify compounds which enhance productivity. (**B**) Rank order plot of 1280 compounds screened on four model cell lines at 3 µM. Fold change in volumetric titer is reported from static cultures. (**C**) Rank order plot of 1280 compounds screened on four model cell lines at 30 µM. Fold change in volumetric titer is reported from static cultures.

To enable screening for enhanced recombinant protein expression in CHO cells, we devised the method illustrated in Figure 1A. We opted to evaluate four independent clones in the small molecule screen to decrease the probability that any identified hits were the result of cell line-specific effects or clonal variability and to increase the applicability of this screen to a wide array of CHO-derived clones and processes. Four independent clonal cell lines were derived from the CHOZN^®^ GS^−/−^ expression platform, two expressing different proprietary IgG_1_ recombinant proteins and two expressing different proprietary Fc-fusion recombinant proteins. Each clone was plated in 96-well plates with media containing a single pharmacologically active small molecule from the LOPAC^®1280^ chemical library, at final concentrations of 3 µM and 30 µM. Cell growth was monitored daily, and volumetric titer was measured after seven days in culture. The fold change in volumetric titer, defined as the amount of antibody produced per given volume of culture, was calculated by dividing the volumetric titer of each individual well of treated cells by the average titer of at least eight replicates of that expressing clone treated with DMSO, which was used to solubilize all the small molecules in the LOPAC^®1280^ library. Of the 1280 small molecules tested, only two molecules, 3F3 (BrdU) and 7A8 (forskolin), enhanced the volumetric titer by a minimum of 1.4× in at least three of the eight conditions tested (4 clonal cell lines × 2 concentrations of each small molecule) (Figure 1B,C and Supplemental Table S1), which corresponds to a hit rate of 0.15% (2/1280 = 0.15%). Interestingly, forskolin has been previously reported to increase recombinant protein production in CHO cells via modulation of both cell cycle and apoptosis pathways [10]. BrdU, on the other hand, has not been previously reported to enhance the production of recombinant proteins, but rather is most recognized as a DNA intercalating agent that can alter DNA stability, prolong the cell cycle, and have effects on transcription and translation [24].

**Figure 2:**
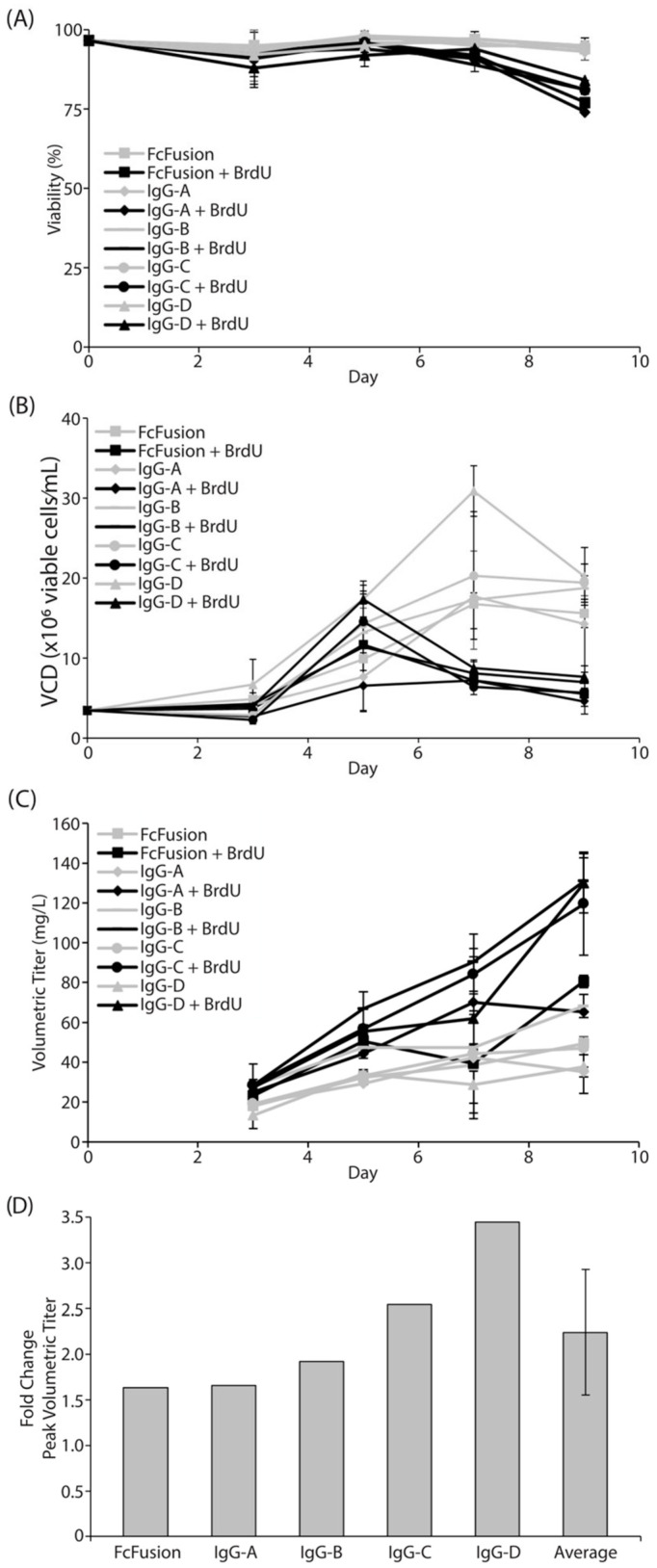
Validation of hit compound BrdU in transient production platform. (**A**) Viability of transiently transfected cells treated with BrdU (black) or untreated (gray). Five independent molecules were transfected into CHOZN^®^ GS^−/−^ cells and viability was measured. Fc Fusion (square), IgG-A (diamond), IgG-B (line), IgG-C (circle), and IgG-D (triangle). (**B**) Viable cell density (VCD) of transiently transfected cells treated with BrdU (black) or untreated (gray). (**C**) Volumetric titer of BrdU-treated (black) and untreated (gray) cells was measured starting on day 3 of a transient productivity assay. (**D**) Fold change in peak volumetric titer of BrdU-treated cultures relative to untreated controls. Average represents the mean peak fold change in the five recombinant proteins tested and indicates consistently higher productivity of BrdU-treated cultures.

To quickly validate the veracity of the LOPAC^®^ library screen results, we utilized transient transfection methodologies to monitor recombinant protein expression in CHO cells. To assure broad applicability of these results to the bioprocessing field and as a result of the less time-consuming nature of transient production processes, four independent IgG_1_ molecules plus one Fc-fusion protein were utilized. Due to the inherent differences of stable and transient protein production processes, we experimentally derived the optimal dose of BrdU to include in transient production processes. A dose of 200 µM provided a balance of culture viability, cell growth, protein production, and reagent cost in a TubeSpin^®^ Bioreactor scale process (Supplemental Figure S1). Using these optimized parameters, we observed a decrease in viable cell density (VCD), a marginal decrease in cell viability, and about a 2× increase in peak protein production in cells treated with BrdU (Figure 2A–C). Across all five recombinant proteins tested, we saw an average increase in peak volumetric titer of 2.2× and a maximum increase of 3.4× (Figure 2D). On the other hand, forskolin did not have a positive impact on transiently transfected cells; however, it is conceivable that through further process optimization, forskolin could provide some benefits in transient transfection systems.

Scaled-down models provide an opportunity to incorporate automation, reduce hands-on time, and lower screening and development costs throughout the drug development life cycle [9]. As a result, we compared the effects of BrdU in a transient production process in TubeSpin^®^ Bioreactors (~20 mL) and 24-deep well plates (~3 mL). As expected, at both scales, we observed an increase in volumetric titer upon treatment with BrdU, suggesting that the effects reported here are scalable (Supplemental Figure S2). Taken together, these results suggest that supplementation with BrdU in cell culture media has a positive impact on the total yield of therapeutic antibodies and antibody-like molecules in transient production processes across multiple volumetric scales.

**Figure 3:**
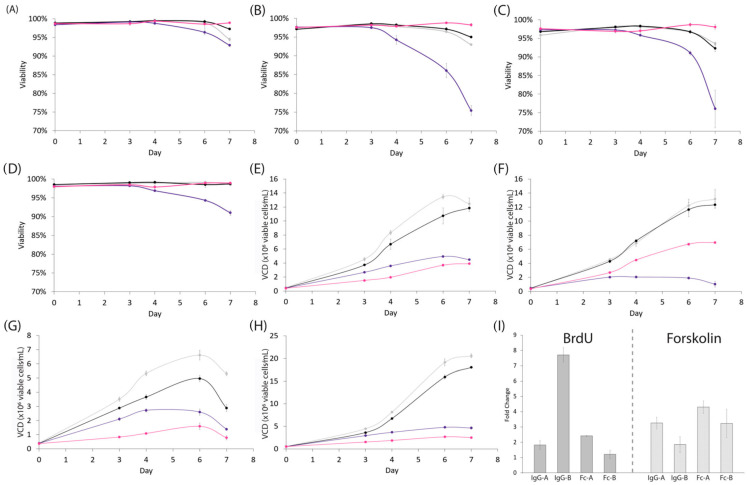
Validation of hit compounds at 30 µM in stably expressing suspension cultures. (**A**–**D**) Viability of stable producing cells in a 7-day batch assay. Cells were treated with DMSO only (black), untreated (gray), 30 µM BrdU (purple), or 30 µM Forskolin (pink). IgG-A (**A**), IgG-B (**B**), Fc Fusion-A (**C**), and Fc Fusion-B (**D**). (**E**–**H**) Viable cell density of stable producing cells in a 7-day batch assay. Cells were treated with DMSO only (black), untreated (gray), 30 µM BrdU (purple), or 30 µM Forskolin (pink). IgG-A (**E**), IgG-B (**F**), Fc Fusion-A (**G**), and Fc-fusion-B (**H**). BrdU and Forskolin both decrease the growth rate of all four clonal cell lines. (**I**) Fold change in qP of cells treated with either 30 µM BrdU or 30 µM forskolin relative to DMSO-treated controls.

While transient protocols provide an advantage to development timelines, the creation of stably expressing cell lines remains the gold standard in the industry. As a result, we validated our observations with forskolin and BrdU in a suspension culture setting, by performing a simple 7-day batch assay on four independently developed clonal CHOZN^®^ GS^−/−^ cell lines with 3 µM and 30 µM of BrdU, forskolin, or the equivalent amount of DMSO supplemented into the cultures at the time of seeding. Untreated control samples were included to compare the impact of DMSO. Cell viability and growth were monitored throughout the assay and cell productivity was measured upon termination of the experiment. DMSO had no impact on cell viability and only a minor effect on the cell growth of a subset of cell lines (Figure 3A–H and Supplemental Figure S3). Unlike previous reports, we did not observe any negative effects on cell viability upon adding up to 30 µM forskolin to the media; however, the addition of 30 µM BrdU negatively impacted the viability of two of the four cell lines tested (Figure 3A–D) [10]. A total of 30 µM of forskolin or BrdU universally reduced the rate of cell growth, with treated samples reaching ≤50% of the peak VCD of the DMSO and untreated cultures (Figure 3E–H). Importantly, 75% of the cell lines tested with forskolin or BrdU showed at least a 2× enhancement in the cell-specific productivity (qP) and a maximal increase of 7.7× in qP compared to DMSO control cultures (Figure 3I). Treatment with 3 µM of either forskolin or BrdU had no impact on cell viability and a cell line-dependent impact on growth rates (Supplemental Figure S3A–H). Two cell lines (IgG-A and IgG-B) exhibited a > 1.5× increase in production when treated with 3 µM BrdU and two cell lines (Fc-A and Fc-B) displayed a ≥1.5× increase in production when treated with 3 µM Forskolin (Supplemental Figure S3I). Our results suggest that maximizing the cellular response to BrdU or forskolin would require the media be supplemented with >3 µM of the respective compound, although we anticipate that further optimization would need to be performed on a clone-by-clone basis, which is beyond the scope of this work.

### 3.2. BrdU Enhances the Efficiency of Recombinant Protein Production in Intensified Processes

As a result of their flexibility, continuity, and cost efficiency, intensified processes have gained significant traction in the bioprocessing field. One challenge of steady-state perfusion processes is assuring that the quantity of drug product lost to cell bleeds, which are used to maintain the biomass at a specific set point, is minimal, because when cells are removed from the culture vessel, the media, which contain valuable drug product, are also directed to the waste stream rather than the harvest tank, ultimately leading to lower yields of the drug product. Given the observation that BrdU slows cell growth in a simple 7-day batch assay (Figure 3E–H), we sought to determine if it would have a comparable effect in a simulated perfusion process, which could result in a less cumbersome method to control VCD and/or biomass, smaller or less frequent cell bleeds, and ultimately a decrease in the loss of precious drug product.

**Figure 4:**
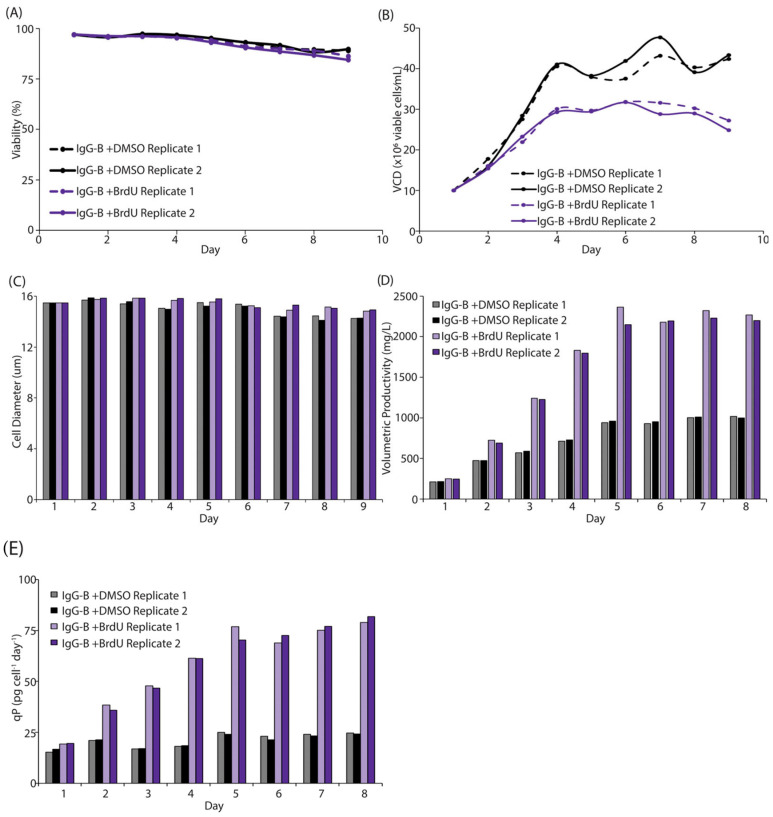
BrdU reduces cell growth and enhances productivity via alterations in the transcriptome. (**A**) Viability of clone IgG-B is comparable when treated with either BrdU (purple) or DMSO (black) in duplicate. (**B**) Viable cell density measurements of two replicates of clone IgG-B treated with BrdU (purple) or DMSO (black). BrdU-treated cultures have a slower growth profile and reach a reduced peak viable cell density. (**C**) Cell diameter of clone IgG-B treated in duplicate with either BrdU (purple) or DMSO (black). BrdU-treated cultures maintain a larger cell diameter for the duration of the assay. (**D**–**E**) Volumetric productivity (**D**) and qP (**E**) of clone IgG-B treated in duplicate with either BrdU (purple) or DMSO (black). Both volumetric productivity and qP increased in BrdU-treated cultures.

The clonal cell line IgG-B was seeded in EX-CELL^®^ Advanced HD Perfusion Media at 1 × 10^7^ viable cells/mL in TubeSpin^®^ Bioreactors with either 1 mM BrdU solubilized in DMSO (1 mM was chosen based on a dose–response curve or DMSO alone. Cell viability, VCD, cell diameter, and IgG titer were monitored, and the full volume of media was exchanged daily for nine days. No significant difference was observed in cell viability and the viability was maintained at ≥90% for the entire duration of the simulated perfusion assay (Figure 4A). Consistent with the pattern observed in the 7-day batch assay, DMSO-only cultures reached a peak VCD of ~4.5 × 10^7^ viable cells/mL, while the BrdU-treated culture reached a peak VCD of only 3.2 × 10^7^ viable cells/mL, a decrease of ~30% relative to the DMSO-only control (Figure 4B). Furthermore, the cells treated with BrdU had a larger cell diameter during the later stages of the assay (Figure 4C). The ~0.8 µm difference in diameter observed in the final three days of the assay amounts to a ~15% increase in the volume of the cell and may provide additional space for important cellular organelles required for energy production, protein synthesis, post-translational modification, and/or protein secretion. In fact, quantification of immunofluorescence staining of the mitochondria indicates there is 4.1× more mitochondrial mass in cells treated with BrdU than cells treated with DMSO (Supplemental Figure S4). Interestingly, it has been reported in other mammalian cell lines, including HEK293 cells, that treatment with small molecules results in decreased growth and an increase in cell size and protein production, similar to what was observed upon treatment of CHOZN^®^ GS^−/−^ cells with BrdU [9]. Akin to the HEK293 cell system, we observed a 2.3× increase, from 1019 mg/L to 2362 mg/L, in the peak daily volumetric titer as well as a 3.3× increase in qP (25 pg/cell/day vs. 82 pg/cell/day) when the stably expressing IgG-B clone was treated with BrdU (Figure 4D,E). Given that BrdU is known to interact with DNA, we sought to determine if there was an increased incidence of mutations in the heavy chain or light chain RNA when cells were treated with BrdU, which could cause significant problems in the development of therapeutic proteins. Importantly, we noted that there was not an increased incidence in mutations in the heavy chain or light chain RNA of BrdU-treated cultures compared to DMSO-treated cultures (Supplemental Figure S5). Taken together, these data suggest that BrdU has a positive impact on the productivity of stably expressing clones in intensified processes; however, due to the inherent limitations of simulated perfusion assays, it does not provide any information regarding the loss of recombinant protein to the cell bleed and minimal information about the metabolic or energetic profile of cells treated with BrdU.

As a result, we performed a similar experiment in the more controlled environment afforded by the Mobius^®^ Breez microbioreactor system. Cells were allowed to reach a steady-state optical density (OD_600_) of ~0.54 and were maintained at this steady state for five days before initiating perfusion of 500 µM BrdU, or an equivalent volume of DMSO, starting on day 9 (Supplemental Figure S6A). Viability was >90% for the duration of the experiment (Supplemental Figure S6A). In the microbioreactor perfused with DMSO, the volumetric productivity and qP of the harvest remained constant at ~200 mg/L/day and ~5 pg/cell/day, respectively. In the microbioreactor perfused with BrdU, the volumetric productivity and qP of the harvest reached ~850 mg/L/day and 30 pg/cell/day, an increase of ~4× and ~6×, respectively (Supplemental Figure S6B). Similar to the simulated perfusion experiment, there was a three-day delay from BrdU administration to the boost in productivity. Finally, while the perfusion rate of both microbioreactors was maintained at ~1.7 vessel volumes per day (VVD), the addition of BrdU resulted in a decrease in the bleed rate from ~0.4 VVD to as low as ~0.01 VVD while increasing the harvest from ~1.2 VVD to nearly the entire 1.7 VVD, effectively allowing all of the recombinant protein to be captured in the harvest and consequently permitting very little to be lost in the cell bleed (Supplemental Figure S6C). This 42% increase in the harvest volume could result in substantial cost savings in a commercial-scale perfusion process.

### 3.3. BrdU Invokes Changes in the CHO Transcriptome Which Can Be Partially Recapitulated via Targeted Genome Engineering

**Figure 5:**
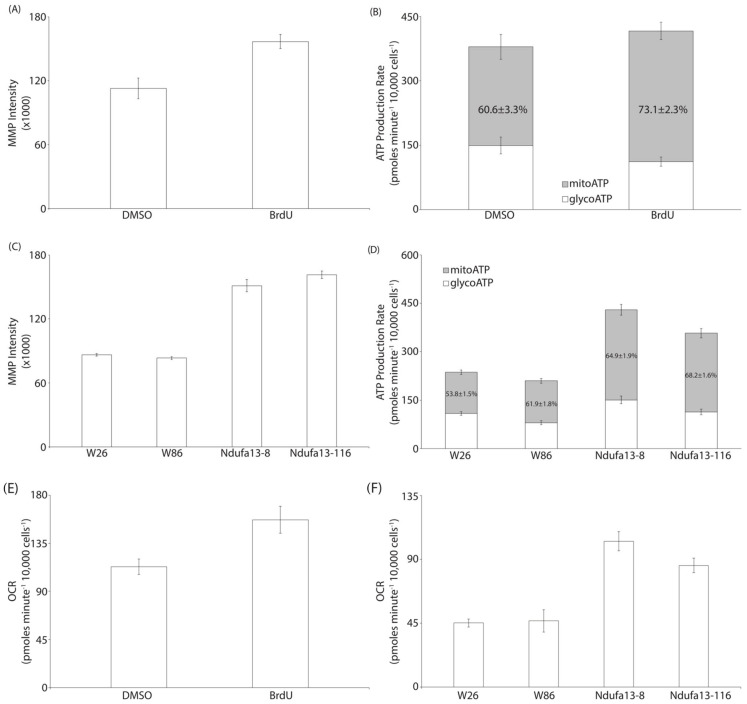
BrdU treatment or genetic modification of Ndufa13 leads to enhanced mitochondrial membrane potential and alters ATP production. (**A**,**C**) MMP was measured via FACS analysis after staining BrdU- and DMSO-treated cells with TMRM (**A**). Additionally, MMP was measured on Ndufa13 edited and wildtype clones (**C**). BrdU treatment as well as genetic modification of Ndufa13 led to increased MMP. (**B**,**D**) ATP production rate as well as the mitochondrial and glycolytic contributions to ATP production were measured in BrdU- and DMSO-treated cells (**B**) and cells with genetic modifications of Ndufa13 as well as identically treated wildtype counterparts (**D**). (**E**,**F**) Oxygen consumption rate (OCR) was measured in BrdU- and DMSO-treated cells (**E**) and cells with genetic modifications of Ndufa13 as well as identically treated unedited counterparts (**F**). BrdU treatment as well as modification of the Ndufa13 gene led to an increased OCR.

While adding BrdU to small-scale cultures is feasible, doing so at a commercial scale may be unrealistic for several reasons, including the additional time and cost required to remove BrdU during downstream processing, assurance that BrdU has no effect on the chemistry or stability of costly purification resins, financial considerations of adding a small molecule to a large bioreactor, and the stability and availability of BrdU as a raw material. To overcome these concerns, we isolated RNA from cells in a simulated perfusion assay and performed RNA sequencing and differential gene expression analysis to determine if the effects of BrdU can be recapitulated via genetic engineering. All gene expression analysis data are available in Supplemental Table S2. The Reactome database was used to identify the top 20 up- and downregulated cellular pathways [19]. Consistent with the decreased growth rate of cells treated with BrdU, we identified numerous cell cycle-related pathways as significantly downregulated. Interestingly, we also identified several mitochondrial and ribosomal pathways, including the mitochondrial electron transport chain complex I, which were downregulated (Table 1). Complex I is the largest protein complex of the mitochondrial electron transport chain and is responsible for the translocation of protons across the inner mitochondrial membrane as well as the transfer of electrons from NADH to coenzyme Q10 during oxidative phosphorylation [25]. As a result of this proton gradient, a potential is created across the mitochondrial membrane and is essential for energy storage during oxidative phosphorylation [26]. Growing evidence suggests that there is a correlation between higher mitochondrial membrane potential (MMP) and increased CHO cell productivity [27,28]. In addition to increased volumetric productivity and qP of cultures treated with BrdU, we observed a 40% increase in the MMP (Figure 5A). Furthermore, we found that cells treated with BrdU generate ~13% more cellular ATP via oxidative phosphorylation and have a 39% higher Oxygen Consumption Rate (OCR) than DMSO-treated counterparts (Figure 5B,E). Together, these results suggest that decreasing the expression of components of Complex I may have a positive impact on the productivity of CHO cells via a shift in their energetic profile. In fact, proteomics analysis of a parental CHO cell line and an MMP-enriched CHO host cell line suggests that several proteins comprising the electron transport chain are present at lower levels in the more productive MMP-enriched host cell line [27].

**Figure 6:**
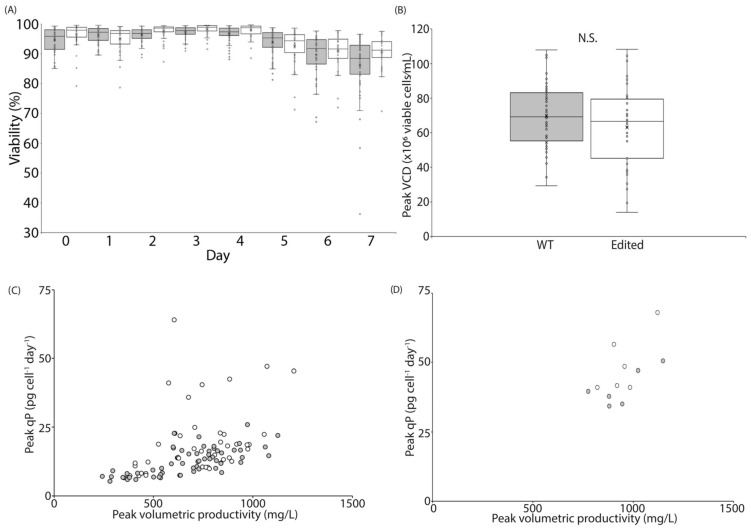
Genetic modification of Ndufa13 partially recapitulates the phenotypes of BrdU treatment. (**A**) The viability of wildtype clones (gray) and Ndufa13 edited clones (white) is similar in a simulated perfusion assay. (**B**) Peak viable cell density of the population of all unedited clones (gray) and all Ndufa13 edited clones (white) was similar. However, many of the top producing Ndufa13 edited clones had slower doubling times. (**C**) The peak volumetric productivity of Ndufa13 edited (white) and wildtype (gray) counterparts is similar. When productivity is normalized by cell number, there is a greater probability of Ndufa13 edited clones having a qP >25 pg cell^−1^ day^−1^. (**D**) The peak volumetric productivity and peak cell-specific productivity of Ndufa13 edited clones treated with BrdU (white) and DMSO (gray).

As an observed effect of BrdU treatment was significantly reduced culture growth, it was reasonable to expect that pathways associated with mitosis, the cell cycle, and even organelle biogenesis would appear in the downregulated Reactome pathway analysis. Indeed, these were among the top enriched pathways; however, as they are likely a result of the growth-suppressive effects rather than causative of the productivity-enhancing phenotype, they were not considered for gene editing efforts. To further reduce the number of targets for genetic engineering, and provided the abundance of mitochondrial processes present in the downregulated pathways, we sought to first investigate cell editing approaches which would attenuate the respiratory functionality of mitochondria. We therefore focused our engineering efforts on Respiratory Complex I, which establishes the proton gradient as the critical first step in cellular aerobic respiration. These combined reasonings reduced the number of genes from several hundred to just thirty-one. Since knockdown of any NDUFS or NDUFV subunit of the Complex I core can be lethal, we excluded these genes and focused instead on those directly involved in catalytic activities [29]. Our final considerations for gene editing targets were three-fold: (1) location within the Complex I structure, (2) *p*-value of gene downregulation, and (3) log2 fold-change rank, with the goal of targeting one gene residing in the mitochondrial matrix, and one gene in the transmembrane portion of Complex I. With these criteria, we identified *Ndufa5* as having the highest degree of downregulation amongst the subunits residing in the mitochondrial matrix [30] (log2 fold-change of −0.654 and *p*-value of 2.31 × 10^−6^), and among the transmembrane subunits, we identified *Ndufb7* and *Ndufa13*. Although *Ndufb7* does exhibit a higher degree of downregulation and statistical significance (log2 fold-change of −0.699, *p*-value of 7.83 × 10^−6^) compared to *Ndufa13* (log2 fold-change of −0.434, *p*-value of 4.22 × 10^−5^) (Supplemental Table S2), we had difficulties designing guide RNA sequences targeting an early exon of *Ndufb7*, as there are only three exons within the gene sequence. Ribonucleoprotein complexes targeting *Ndufa13* were transfected into the Fc-fusion-B clone, and transfected cells were then sub-cloned and amplicon sequencing was performed to identify those subclones with nonsense mutations. Interestingly, of the 44 edited clones, none contained nonsense mutations in both alleles, suggesting that *Ndufa13* is essential for CHO cell survival (Supplemental Table S3). This is not surprising given the importance of the electron transport chain as well as the severity of human diseases associated with mutations in *Ndufa13* [30,31]. All 44 edited clones as well as 58 wildtype (WT) clones, in which no edited alleles were detected via amplicon sequencing, were scaled up and subjected to a simulated perfusion assay. No difference was observed in the viability or peak VCD of the WT and edited clones (Figure 6A,B); however, it was noted that many of the edited clones with the highest qP reached a lower peak VCD (Supplemental Table S3). Importantly, eight of the top ten clones, as ranked by peak qP, came from the *Ndufa13* edited clones (Figure 6C). It is feasible that the enhanced performance observed in the *Ndufa13* clones is unrelated to the effects of BrdU treatment. To test the hypothesis that BrdU acts in part through modification of *Ndufa13* activity, we treated six of the top performing *Ndufa13* edited clones with BrdU or an equivalent volume of DMSO. If BrdU acts in part through the electron transport chain, we would expect to see a diminished increase in performance of the BrdU-treated *Ndufa13* edited clones relative to the DMSO-treated controls. On the other hand, if BrdU does not act through the electron transport chain, then we would expect to see a similar enhancement in productivity upon treatment with BrdU. In fact, *Ndufa13* edited clones treated with BrdU showed on average a ~22% increase in qP and a ~2% increase in volumetric productivity (Figure 6D). While a ~22% increase in qP is notable, it is drastically reduced relative to the differences we observed with cell lines assessed in simulated perfusion and the Mobius^®^ Breez microbioreactor system (Figure 4D–E and Supplemental Figure S6). Furthermore, when we genetically disrupted another gene encoding an electron transport chain Complex I component, *Ndufa5*, in clone Fc-fusion-A, we again observed enhanced productivity (Supplemental Table S3). In fact, all of the *Ndufa5* knockout clones outperformed their wildtype counterparts in a simulated perfusion assay. Together, this shows that genetic manipulation of two independent electron transport chain-encoding genes, in two independently derived CHO clones, expressing two independent proprietary Fc-fusion proteins, results in a similar phenotypic change, namely an increase in productivity. Altogether, this suggests, as previously reported, that the reduction in the expression of electron transport chain genes has a positive effect on the productivity of CHO cells [27].

To determine if the mitochondrial phenotypes observed in BrdU-treated cultures were recapitulated in the *Ndufa13* edited clones, we measured the mitochondrial mass, MMP, OCR, total ATP production rate, and the relative contributions of oxidative phosphorylation and glycolysis to ATP production in the top two WT and *Ndufa13* edited clones, as determined by qP and respective growth profiles. We observed a 1.8× increase in mitochondrial mass and MMP of *Ndufa13* edited clones relative to WT clones (Figure 5C and Supplemental Figure S4B). Furthermore, we found that *Ndufa13* edited clones produce 1.8× more ATP than their WT counterparts (Figure 5D) and, similar to BrdU treatment conditions, we observed a 2.1× increase in the oxygen consumption rate (OCR) of *Ndufa13* edited clones relative to their WT counterparts (Figure 5F). Correspondingly, in the Mobius^®^ Breez microbioreactor system, we observed similar growth, viability, and productivity phenotypes to those exhibited in the simulated perfusion assay (Supplemental Figure S7A,B). Additionally, we observed increased oxygen consumption in *Ndufa13* edited clones, as indicated by the reduced oxygen concentration in these bioreactors, suggesting that a higher inflow of compensatory oxygen was required to maintain the 40% oxygen set point (Supplemental Figure S7C). Taken together, this indicates that the bioprocessing parameters associated with BrdU treatment, including growth and productivity, as well as the altered energetics profile, including mitochondrial mass, MMP, OCR, and ATP production characteristics, can be partially recapitulated via genetic engineering of the electron transport chain component *Ndufa13*. Interestingly, in the Mobius^®^ Breez microbioreactor system, we did not observe any alteration to the bleed- or harvest rates of the *Ndufa13* edited clones, suggesting that alterations within the electron transport chain are not responsible for all phenotypes associated with BrdU treatment.

## 4. Discussion

Due to the growing and aging global population, there is a heightened urgency to produce therapeutic proteins. This growing demand requires that either the biomanufacturing community invest significant financial resources into increasing production capacity of manufacturing plants, or that the scientific community develop new technologies capable of increasing therapeutic protein production. While CHO cells are the cell line of choice in producing therapeutic proteins, they have remained largely unchanged since the genetic engineering of glutamine synthetase as a selection system [18,27]. Several high-throughput screens have been performed using small molecule libraries to enhance the performance of CHO cells; however, process economics often precludes incorporating these small molecules into commercial-scale therapeutic protein production settings [3,8,9].

We have overcome these challenges by leveraging a small molecule screen to identify cellular pathways which enhance the productivity of CHO cells. Through this screen, we identified two small molecules, forskolin and BrdU, which enhance the expression of multiple clonal cell lines by ≥40%. Notably, forskolin has been previously reported to enhance the productivity of CHO cells by modulating proliferation and/or apoptosis [10]. To ascertain the molecular mechanisms associated with enhanced productivity of CHO cells exposed to BrdU, we performed RNA-Seq. Cellular pathway analysis of the differential gene expression profile suggests several key pathway changes. Consistent with the reduced growth rate upon treatment with BrdU, we saw enrichment of several cell cycle-related Reactome pathways with reduced expression (separation of sister chromatids, mitotic anaphase, mitotic metaphase and anaphase, M phase, cell cycle/mitotic, cell cycle, and mitotic prometaphase). In addition, we observed reduced expression of energetics pathways (respiratory electron transport, respiratory electron transport/ATP synthesis by chemiosmotic coupling, Complex I biogenesis, the citric acid (TCA) cycle, and respiratory electron transport). To determine if these pathway changes influenced mitochondrial performance, we measured the mitochondrial mass, MMP, the cellular ATP production rate, as well as the contributions of oxidative phosphorylation and glycolysis to ATP production. Interestingly, we found that the MMP of BrdU-treated cells was increased and that ~13% more energy was produced via mitochondrial processes upon BrdU treatment.

Recent work has suggested that cells with increased MMP have enhanced biotherapeutic protein production capacity [27,28]. While these studies focused on selecting cells with high MMP via FACS-based enrichment strategies, it is feasible that we may engineer these favorable MMP traits into CHO cell lines through genetic modification of the cellular pathways identified within our RNA-Seq data, thereby increasing the likelihood of obtaining high-producing clones without the need for additional screening or FACS-based assays. Complex I is the largest protein complex of the mitochondrial electron transport chain and is responsible for the translocation of protons across the inner mitochondrial membrane [25]. By generating this proton gradient, the necessary electrochemical potential is created for energy access during oxidative phosphorylation [26]. Interestingly, Complex I biogenesis was significantly enriched in our Reactome pathway analysis of downregulated genes, and we therefore sought to genetically modulate *Ndufa13* and *Ndufa5*, two components of Complex I. Upon examining sequencing data, we were unable to identify *Ndufa13* clones with nonsense mutations in both alleles, suggesting that it is essential for CHO cell survival; however, we were able to identify *Ndufa5* knockout clones. When comparing the productivity of *Ndufa13* clones with a single edited allele to unmodified counterparts, we observed that eight of the top ten producing clones, as determined by qP, came from the edited population. Likewise, all of the *Ndufa5* knockout clones outperformed their wildtype counterparts in a simulated perfusion assay, as measured by qP. Further analysis of the top two *Ndufa13* edited and unedited clones, as determined by qP with comparable growth profiles, showed that the edited clones had higher MMP as well as a shift in their ATP production profiles, similar to cells treated with BrdU. It has been suggested that MMP-enriched CHO cells may have a higher turnover of mitochondria, resulting in an improvement in mitochondrial function [27]. Likewise, it is conceivable that the increased mitochondrial ATP production that we observed in *Ndufa13* edited cells is a result of increased mitochondrial turnover which, as was reported in MMP-enriched CHO cells, could lead to increased mitochondrial ATP production and ultimately higher recombinant protein yields. Alternatively, it is plausible that the increased mitochondrial ATP production that we observed in *Ndufa13* edited cells is a result of an increase in mitochondrial biogenesis, which is supported by our observation that there was a substantial increase in the mitochondrial mass of *Ndufa13* edited clones as well as BrdU-treated cultures. While our data strongly suggest that BrdU treatment of CHO cells has a significant impact on mitochondrial function, the balance of energy production between glycolysis and oxidative phosphorylation and enhanced bioprocessing phenotypes, it is still conceivable that a combination of effects that were identified via the differential gene expression analysis and previously published work, including effects on the cell cycle, transcription, translation, and DNA stability, also plays a role in the phenotypes reported here [24].

In conclusion, we identified two small molecules which increased the productivity of clones expressing a diverse array of monoclonal antibodies and Fc fusion proteins then translated the effects of one of these molecules, BrdU, into genetic targets which partially recapitulate the phenotypes associated with BrdU treatment. Our RNA-Seq results suggest that the effects of BrdU are complex and impact multiple cellular pathways, making it unlikely that modification of a single gene or pathway would fully recapitulate the effects of BrdU treatment. However, the findings described here could provide a blueprint to enhance not just the productivity of CHO cells, but other important aspects associated with a favorable bioprocessing host cell line. For example, the process through which a single vial or bag of cells is thawed and expanded for manufacturing processes can take weeks, or even months, depending on the scale. To shorten these timelines, one could employ a similar process to those described here, to identify genetic targets capable of reducing cell doubling times, thereby reducing the scale-up timeline. As was noted in our work, many of the top producing clones have slower growth rates; however, it is possible that employing a combinatorial approach of genetic engineering could restore the growth rate and ensure that these engineered cell lines do not compromise manufacturing schedules. It is also plausible that the contrasting, and potentially incompatible, phenotypes of growth and productivity could be engineered into the cells via synthetic switches, allowing for a seamless transition between fast growth rates and increased productivity. Taken together, we believe the data and methods shared here will lay the foundation for the gene target discovery work required for such a system to be incorporated. Ultimately, this work could play a key role in reducing manufacturing costs and making medications more affordable and accessible to the patients who depend on them.

## Figures and Tables

**Table 1:** RNA-Seq and differential gene expression analysis was performed on samples from day 4 of the simulated perfusion assay using cells treated with either BrdU or DMSO as a control. The top 20 downregulated and top 20 upregulated Reactome pathways are shown.

Regulation	Reactome Pathway	*p*-Value	Number of Genes Up- or Down-Regulated	Number of Genes in Pathway	Percent of Genes Up- or Down-Regulated
Up	Assembly of collagen fibrils and other multimeric structures	−11.41	21	25	84.0%
Up	Extracellular matrix organization	−10.62	77	116	66.4%
Up	Muscle contraction	−9.98	60	79	75.9%
Up	Striated Muscle Contraction	−9.61	15	17	88.2%
Up	Collagen chain trimerization	−9.29	19	19	100.0%
Up	Collagen formation	−8.93	26	40	65.0%
Up	Platelet homeostasis	−8.35	21	28	75.0%
Up	Cross-presentation of particulate exogenous antigens (phagosomes)	−8.15	6	6	100.0%
Up	Nitric oxide stimulates guanylate cyclase	−7.37	8	8	100.0%
Up	Class A/1 (Rhodopsin-like receptors)	−7.13	54	74	73.0%
Up	SLC-mediated transmembrane transport	−6.83	91	139	65.5%
Up	Peptide ligand-binding receptors	−6.72	29	43	67.4%
Up	Transport of inorganic cations/anions and amino acids/oligopeptides	−6.63	43	59	72.9%
Up	Collagen degradation	−6.59	10	15	66.7%
Up	Dopamine Neurotransmitter Release Cycle	−6.29	5	6	83.3%
Up	Hemostasis	−6.24	169	252	67.1%
Up	Transmembrane transport of small molecules	−6.11	194	313	62.0%
Up	Collagen biosynthesis and modifying enzymes	−6.10	22	32	68.8%
Up	cGMP effects	−6.01	7	7	100.0%
Up	Li1CAM interactions	−5.56	34	52	65.4%
Down	Mitochondrial translation termination	−56.18	58	63	92.1%
Down	Mitochondrial translation	−55.45	58	63	92.1%
Down	Mitochondrial translation elongation	−52.86	55	60	91.7%
Down	Organelle biogenesis and maintenance	−48.58	143	205	69.8%
Down	Gene Expression	−32.19	444	721	61.6%
Down	Separation of Sister Chromatids	−25.58	85	115	73.9%
Down	Mitotic Anaphase	−25.37	89	121	73.6%
Down	Mitotic Metaphase and Anaphase	−25.12	90	122	73.8%
Down	M Phase	−22.21	119	166	71.7%
Down	Cell Cycle, Mitotic	−22.16	193	312	61.9%
Down	Respiratory electron transport	−20.17	37	41	90.2%
Down	Repiratory electron transport, ATP synthesis by chemiosmotic coupling, and heat	−19.06	46	53	86.8%
Down	Metabolism of proteins	−18.39	344	630	54.6%
Down	Complex I biogenesis	−17.69	28	31	90.3%
Down	Processing of Capped Intron-Containing Pre-mRNA	−17.27	110	157	70.1%
Down	Cell Cycle	−16.08	45	89	50.6%
Down	Post-translational protein modification	−15.93	272	516	52.7%
Down	DNA Repair	−15.38	115	187	61.5%
Down	The citric acid (TCA) cycle and respiratory electron transport	−15.12	62	80	77.5%
Down	Mitotic Prometaphase	−15.00	54	76	71.1%

## Data Availability

The data that support the findings of this study are available from the corresponding author upon reasonable request.

## Supplementary Materials

The following supporting information can be downloaded at: https://www.mdpi.com/article/10.3390/cells12222661/s1, Figure S1: BrdU dose optimization in transient transfection process; Figure S2: BrdU transient transfection is consistent across scales; Figure S3: Validation of hit compounds at 3 µM in stably expressing suspension cultures; Figure S4: BrdU treatment or genetic modification of Ndufa13 leads to enhanced mitochondrial mass; Figure S5: Treatment of CHO cells with BrdU has no effect on the mutation rate of the heavy chain or light chain RNA; Figure S6: Perfusing BrdU into a Mobius^®^ Breez microbioreactor system leads to enhanced productivity and decreased cell bleeds; Figure S7: Ndufa13 edited clones have higher productivity and increased oxygen demands in a Mobius^®^ Breez microbioreactor system; Table S1: LOPAC®1280 cell specific productivity fold change; Table S2: RNA-Seq differential gene expression analysis; Table S3: Genotypes and simulated perfusion performance of Ndufa13 edited and Ndufa5 knockout clones.
