# Supplementary figures and images for: Chemical and Genetic Modulation of Complex I of the Electron Transport Chain Enhances the Biotherapeutic Protein Production Capacity of CHO Cells
Source: Cells. 2023 Nov 20;12(22):2661. doi: 10.3390/cells12222661 (PMC10670226; doi:10.3390/cells12222661)

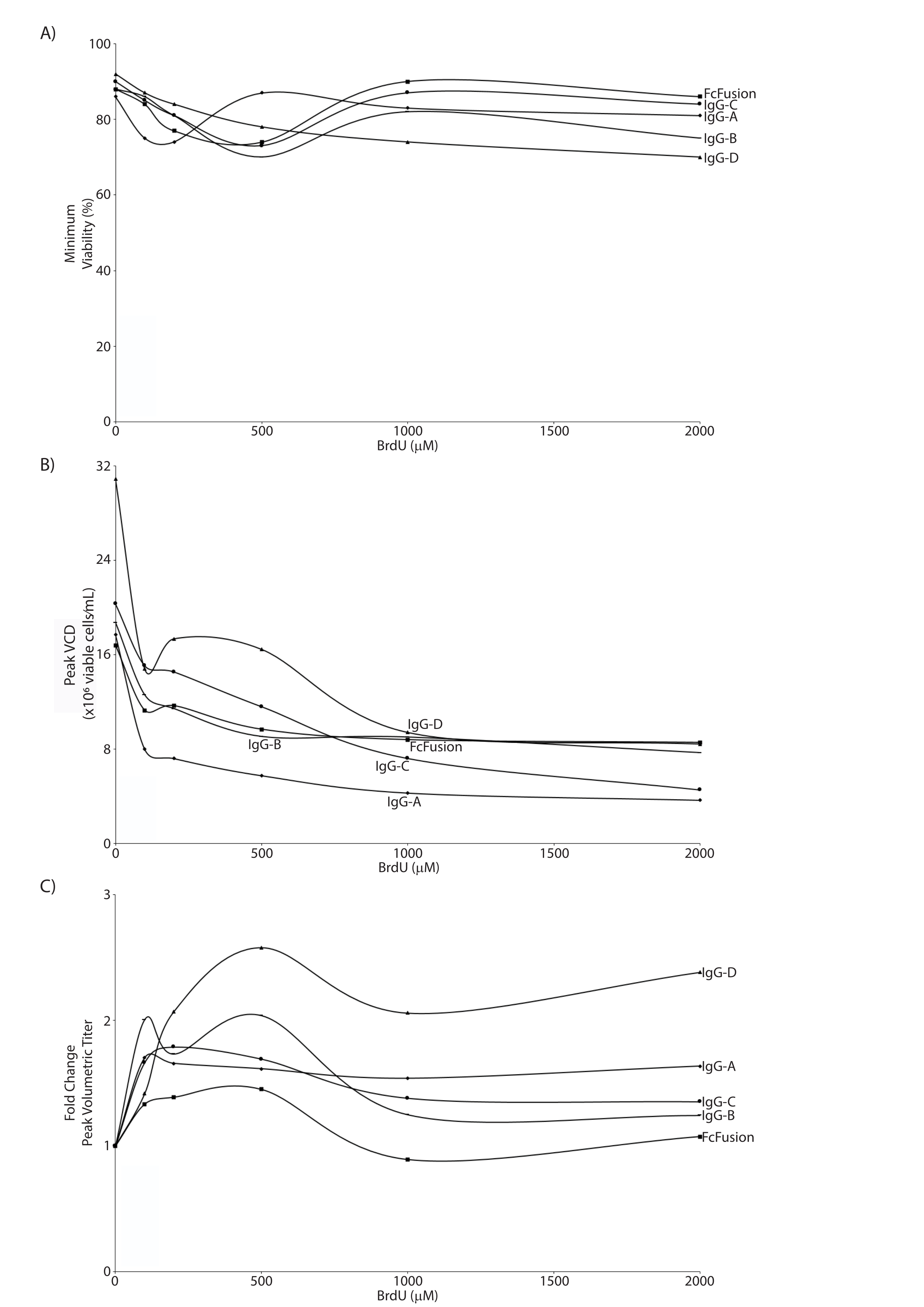

Supplement: Supplementary file 1 [file cells-12-02661-s001.zip › Supplemental Figure 1.png]

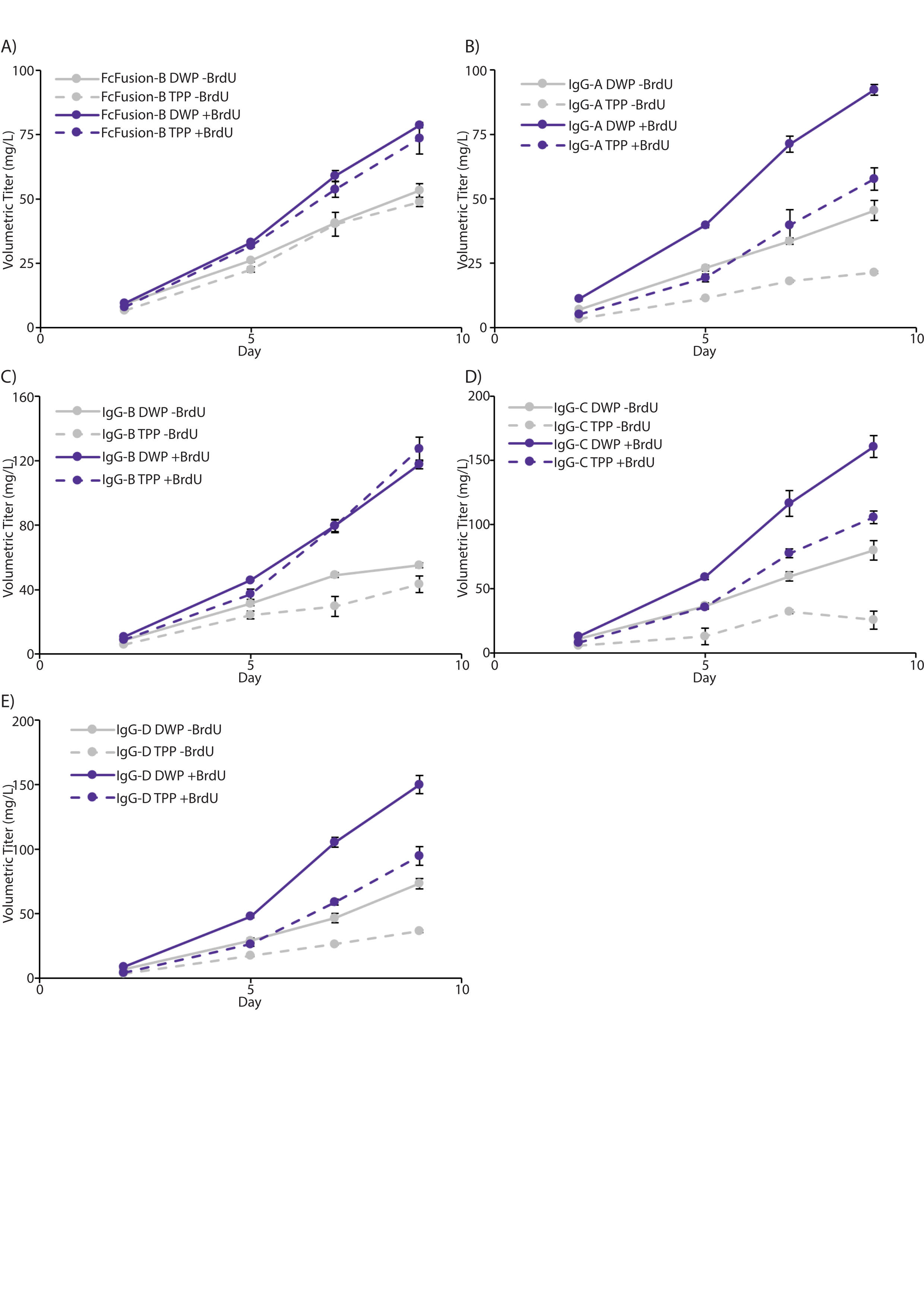

Supplement: Supplementary file 1 [file cells-12-02661-s001.zip › Supplemental Figure 2.png]

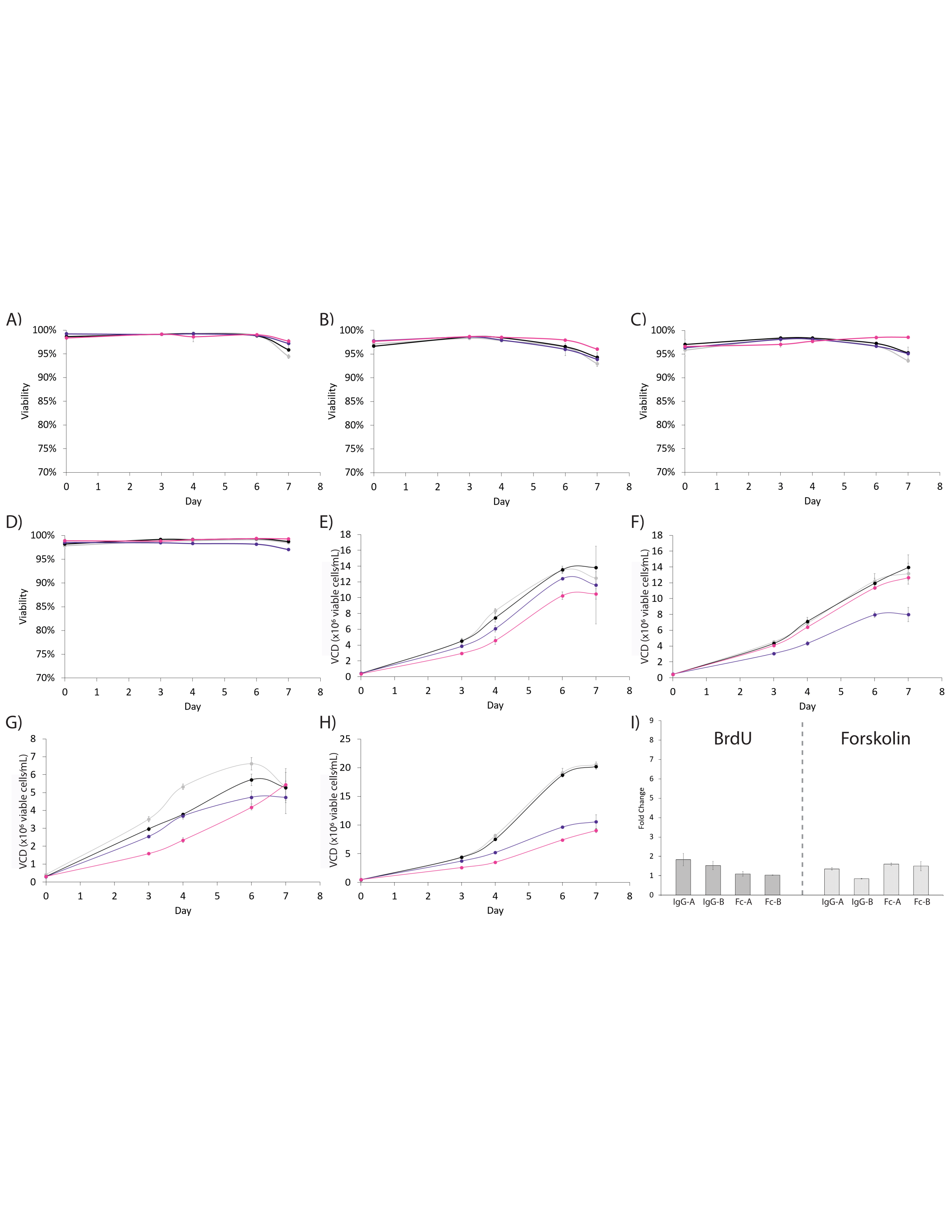

Supplement: Supplementary file 1 [file cells-12-02661-s001.zip › Supplemental Figure 3.png]

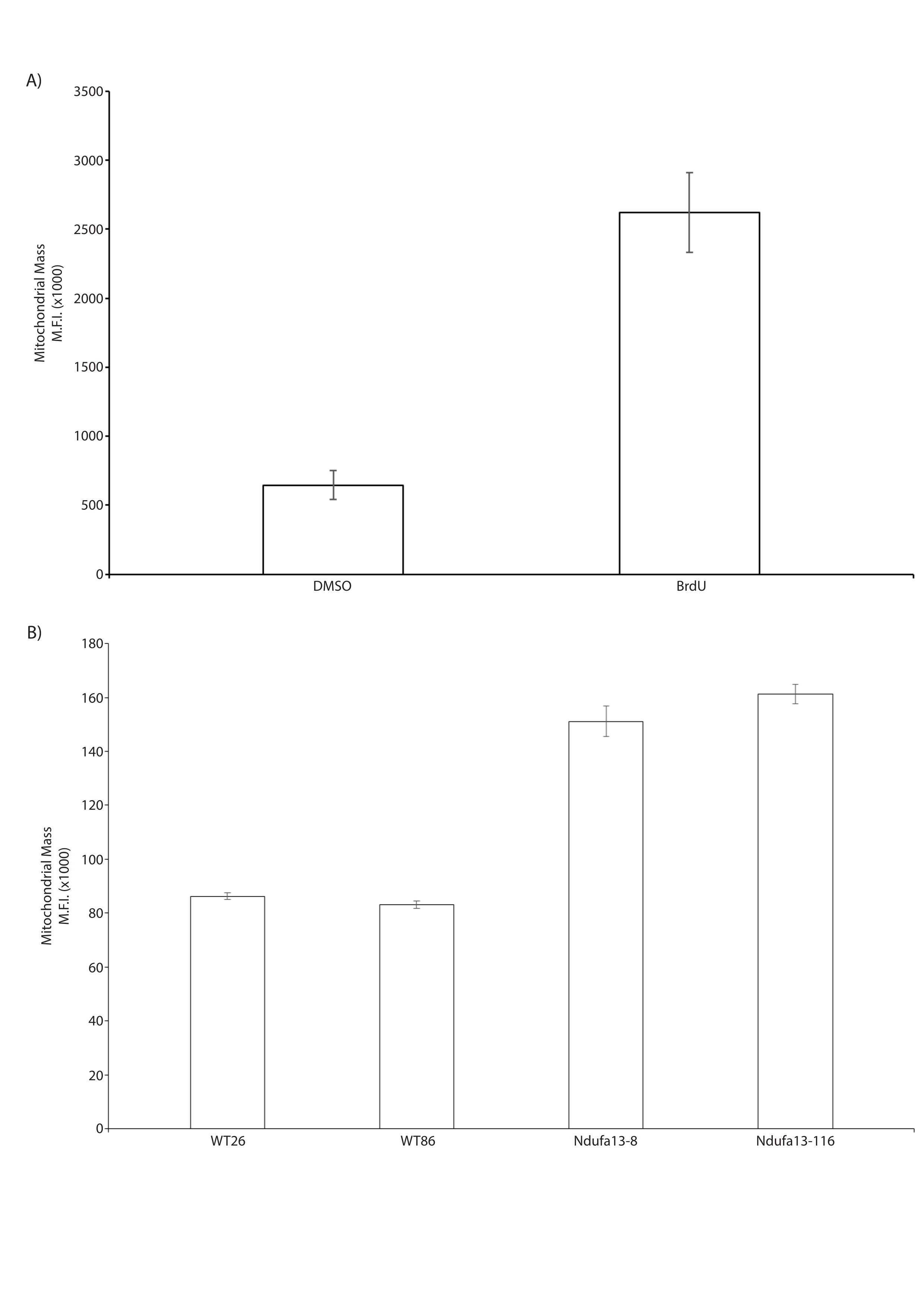

Supplement: Supplementary file 1 [file cells-12-02661-s001.zip › Supplemental Figure 4.png]

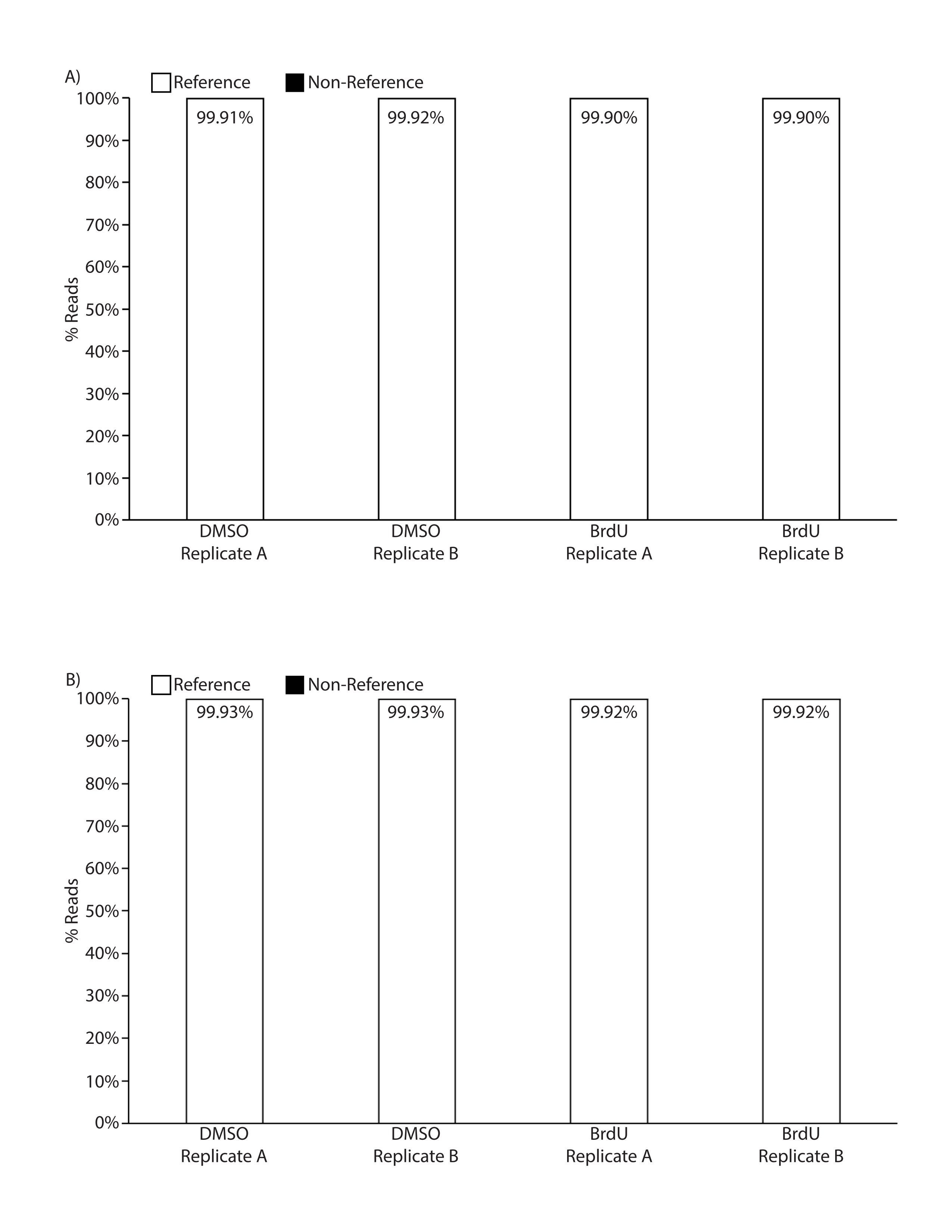

Supplement: Supplementary file 1 [file cells-12-02661-s001.zip › Supplemental Figure 5.png]

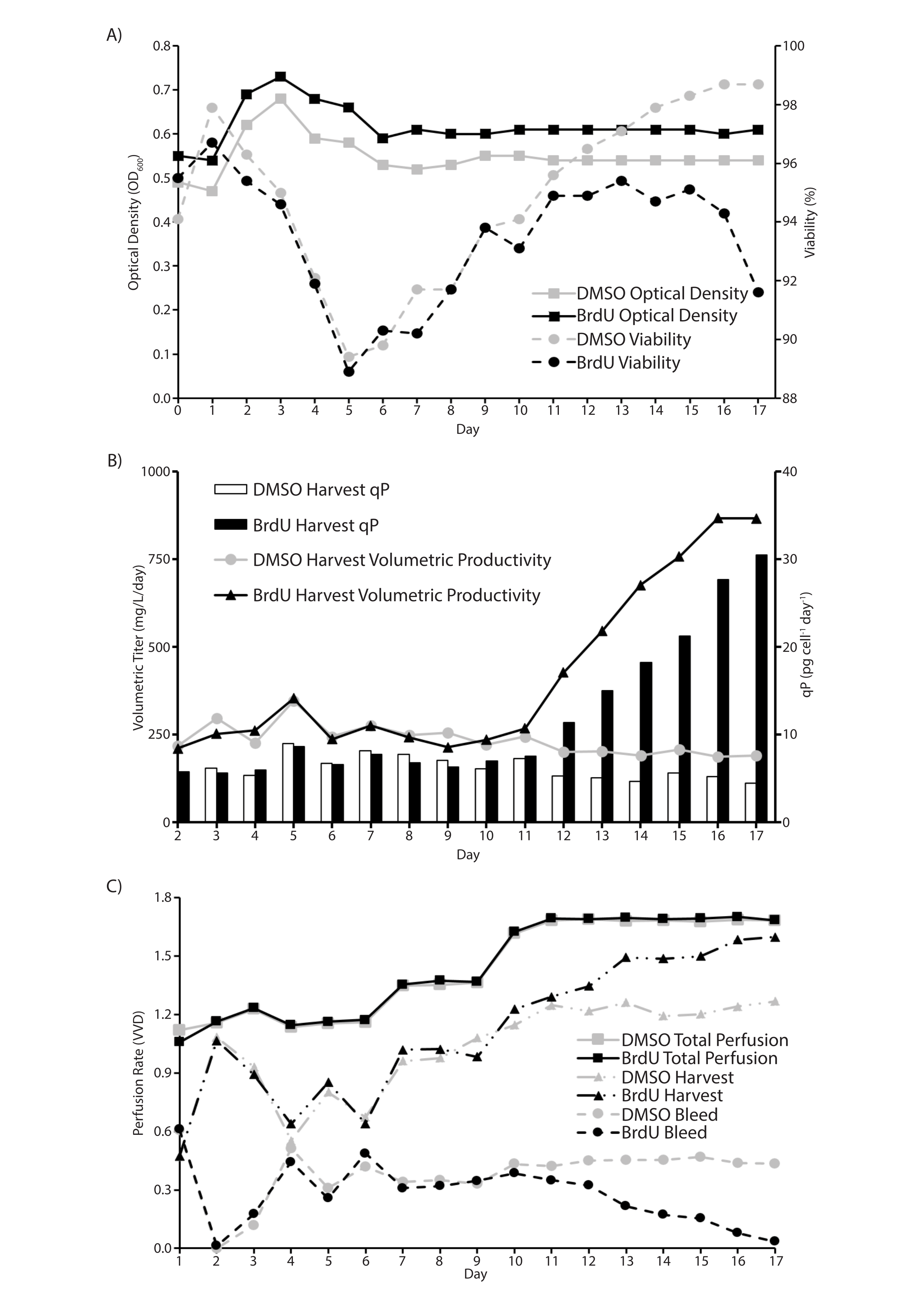

Supplement: Supplementary file 1 [file cells-12-02661-s001.zip › Supplemental Figure 6.png]

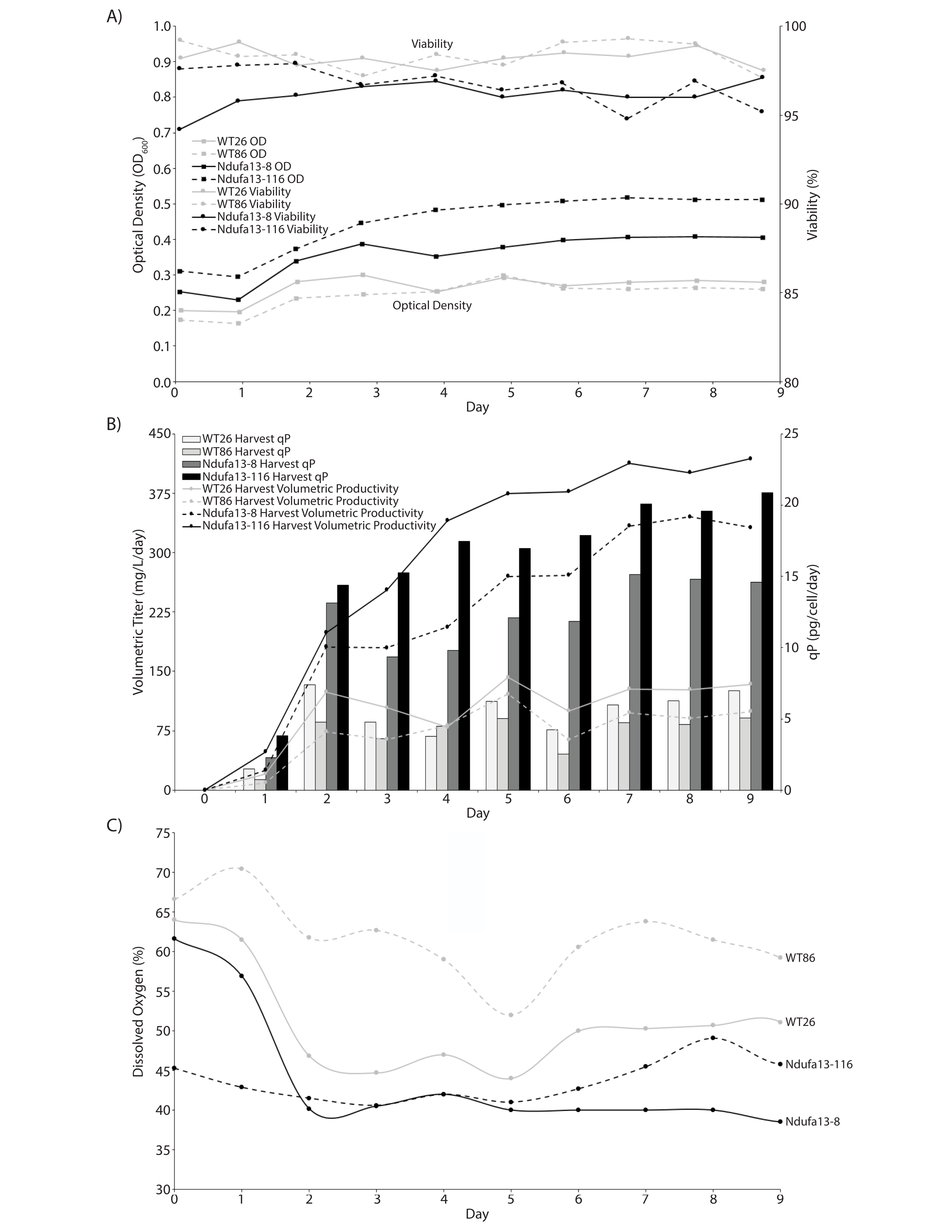

Supplement: Supplementary file 1 [file cells-12-02661-s001.zip › Supplemental Figure 7.png]
